# Oxygen sensing; a stunningly elegant molecular machinery highjacked in cancer

**DOI:** 10.1080/03009734.2020.1769231

**Published:** 2020-06-24

**Authors:** Lena Claesson-Welsh

**Affiliations:** Department of Immunology, Genetics and Pathology, Rudbeck Laboratory, Uppsala University, Beijer, and SciLifeLab Laboratories, Uppsala, Sweden

**Keywords:** Cancer, hypoxia, prolyl hydroxylation, VEGF

## Abstract

Oxygen is of fundamental importance for most living organisms, and the maintenance of oxygen homeostasis is a key physiological challenge for all large animals. Oxygen deprivation, hypoxia, is a critical component of many human diseases including cancer, heart disease, stroke, vascular disease, and anaemia. The discovery of oxygen sensing provides fundamental knowledge of a stunningly elegant molecular machinery; it also promises development of new therapeutics for serious diseases such as cancer. As a result of their impressive contributions to our understanding of the mechanisms by which cells sense oxygen and signal in hypoxia, Gregg Semenza, Peter Ratcliffe, and William Kaelin were awarded the Nobel Prize in 2019.

## Introduction

**Figure 1.**
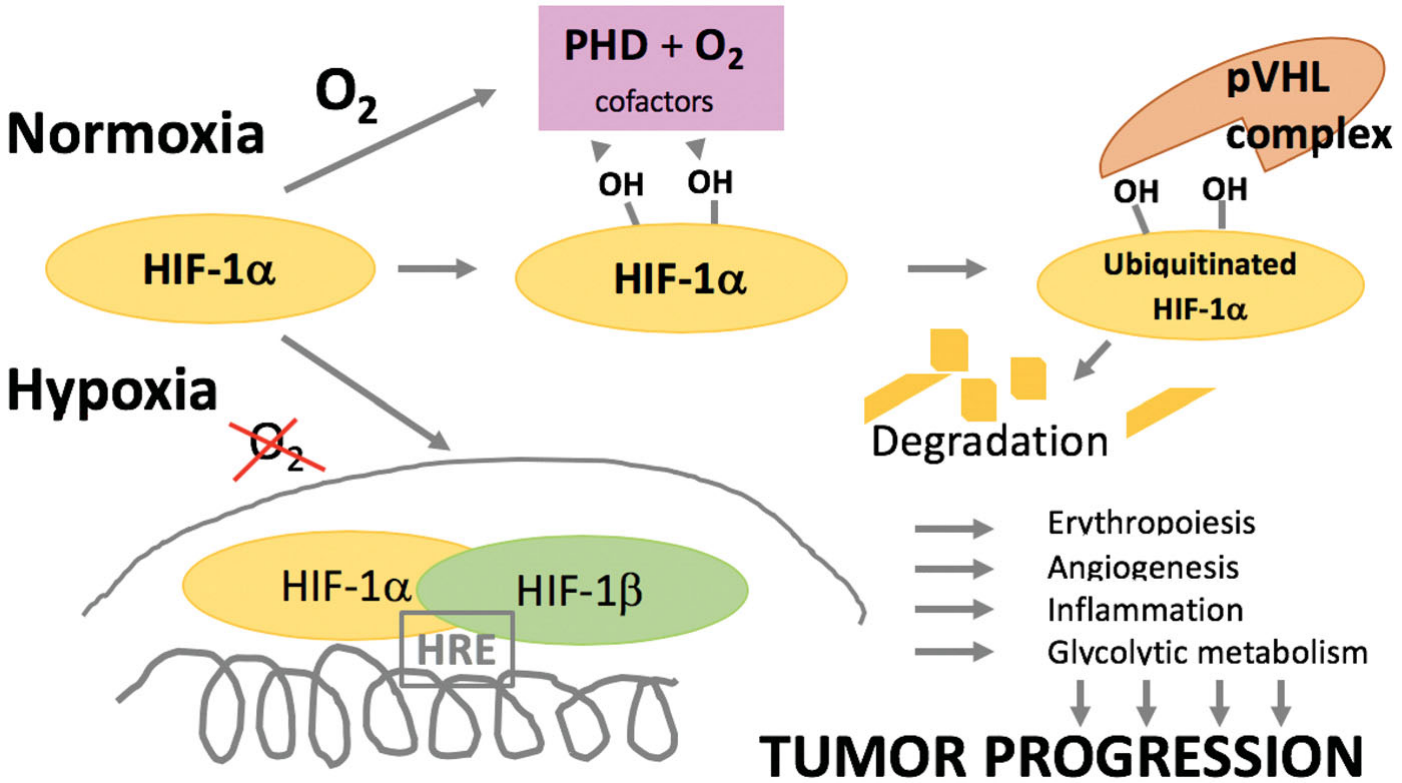
Simplified schematics of oxygen sensing and transcriptional regulation. In normoxia the hypoxia-inducible factor (HIF)-1α subunit becomes hydroxylated on several residues by prolyl hydroxylase (PHD), which uses molecular oxygen as a substrate. Hydroxylated HIF-1α is recognised by the von Hippel Lindau (pVHL) complex which catalyses its ubiquitination and degradation. In hypoxia, HIF-1α remains stable and translocates to the nucleus to form a complex with the HIF-1β subunit to become a transcriptionally active complex, binding to the hypoxia-responsive element (HRE). Genes are induced that regulate a wide range of processes exploited to serve the progression of cancer.

How is oxygen tension sensed, and what is the consequence of oxygen sensing? The oxygen-sensitive signal is generated by enzymes that catalyse hydroxylation of specific prolyl and asparaginyl residues in hypoxia-inducible factor (HIF). HIF is the key transcription factor that regulates transcriptional responses to hypoxia. Hydroxylation of HIF at different phylogenetically conserved sites targets it for degradation in normoxia. In hypoxia, HIF escapes destruction and forms active transcriptional complexes that control expression of thousands of genes in the human genome. HIF is a heterodimer consisting of one α (oxygen-sensitive) and one β (oxygen-insensitive) subunit. There are three α subunits with partly overlapping but also distinct functions. Many of the most prominent and well-characterized HIF-regulated genes have key functions in oxygen supply and utilisation via erythropoiesis, angiogenesis, haematopoiesis, and metabolic reprogramming (1). In order to coordinate the most efficient use of oxygen by the cell, HIFs activate genes that shift energy dependence away from high oxygen demand, towards glycolysis. The genes encoding essentially all glycolytic enzymes are directly upregulated by HIFs (2,3). In addition to pathways important for maintaining oxygen homeostasis, HIF targets are involved in autophagy, apoptosis, redox homeostasis, inflammation and immunity, stemness and self-renewal, and metastasis and invasion (Figure 1) (2,4–6).

Below follows an outline of the scientific careers and the contributions of Gregg L. Semenza, Peter J. Ratcliffe, and William G. Kaelin to our understanding of oxygen sensing. It is very interesting to read the bibliographies of these outstanding scientists as they cover the entire development of the field. Moreover, it is particularly impressive that all three, Semenza, Ratcliffe, and Kaelin, have been clinically active at least during parts of their research careers. This may serve as an inspiration for physicians today, to use their deep clinical insights as a foundation for excellent research. It is indeed possible, and important, to engage in both clinical work and in science, and to do both at a top level!

## Gregg L. Semenza


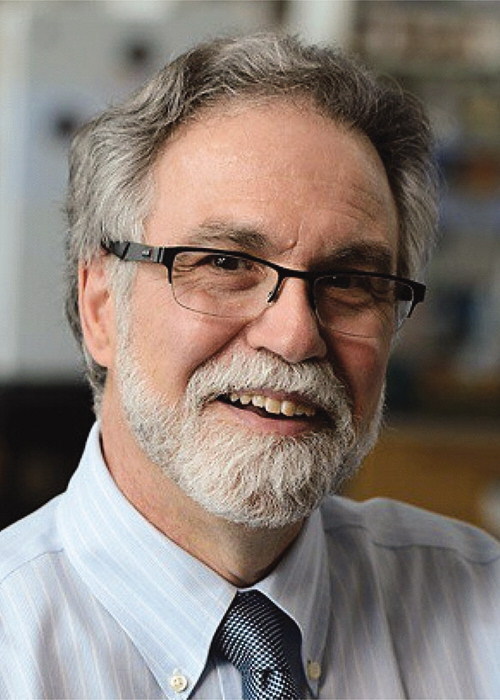


After graduating from Harvard, Gregg L. Semenza joined the MD/PhD programme at University of Pennsylvania. For his PhD, he studied β-thalassemia. He did his residency in paediatrics at Duke University. In 1986, Semenza started his postdoc training in medical genetics at Johns Hopkins School of Medicine, first working on haemophilia but later shifting his focus to erythropoietin (EPO) and developmental regulation of EPO expression, which was known to switch from foetal liver to adult kidney. The goal was to identify DNA sequences controlling organ-specific EPO expression (7). This work led to the identification of the hypoxia-responsive element (HRE), regulating expression of the EPO gene (8). In parallel, Peter Ratcliffe’s group (9) as well as other groups (10) had identified the same or overlapping sequences in the mouse and human EPO genes. Clearly, these studies represented a major breakthrough in the understanding of hypoxia regulation.

Semenza and colleagues went on to identify HRE sequences in a wide range of genes implicated in O_2_ homeostasis (11). The HRE was narrowed down to 33 nucleotides and shown to bind a hypoxia-induced nuclear factor (12). In a subsequent study published in 1993, Semenza mentions HIF-1 for the first time and shows that it binds to DNA also in cells not normally expressing EPO. This work led to the conclusion that HIF-1 and its recognition sequence are common components of a general mammalian cellular response to hypoxia (13).

Semenza went on to show the oxygen-regulated, rapid kinetics of HIF-1 binding to DNA (14). Subsequently, his group reported on the purification of HIF-1 which was identified as a heterodimer composed of HIF-1α and HIF-1β, with the ability to bind to an intact but not mutated HRE (15). In 1995, Semenza and co-workers in their elegant and very impressive hallmark paper (16) deduced the structure of the HIF-1 subunits as basic-helix-loop-helix proteins containing a PAS (Per-ARNT-AHR-Sim) domain. This was done by sequencing tryptic peptides from the purified HIF preparation, and this information was used as the basis for cDNA cloning. In their outstanding paper, his group also demonstrated that HIF-1, at both RNA and protein levels (using newly generated antibodies), was induced in cells exposed to 1% O_2_ but that they decayed remarkably rapidly upon return of the cells to 20% O_2_ (16). They moreover presented indications for posttranslational modification of HIF-1α.

In 1997, Semenza implicated HIF-1 in tumorigenesis for the first time (17). By generating HIF-1α knockout mice, the essential role for HIF-1α in embryonic development was demonstrated. Lack of HIF-1α in mice resulted in lethality at E11 with neural tube defects and cardiovascular malformations (18). The authors proposed that the time point of lethality coincided with that of the embryo size exceeding what could be oxygenated by passive diffusion from the mother’s circulation.

In 2000, the Semenza lab addressed oxygen-dependent ubiquitination in HIF-1α regulation (19). Semenza went on to describe FIH-1 (factor inhibiting HIF-1) as a protein binding to and negatively regulating the C-terminal transactivation domain of HIF-1α (20). FIH-1 was subsequently shown by Ratcliffe and others to be an asparaginyl hydroxylase (see below).

Semenza then largely turned to the role of hypoxia in diseases. One particular interest was the role of the hypoxia-regulated vascular endothelial growth factor (VEGF) and dysfunctional angiogenesis in cancer (21)—a research direction shared with Ratcliffe (22) and Kaelin (see below). Together with others, the Semenza group identified overexpression of HIF-1 in many cancer types and in metastases (23). In cancer, VEGF-regulation through HIF-1 involves increased HIF-1 expression rather than regulation of its half-life (24). A major focus for Semenza has thereafter been to screen for HIF inhibitors, which has led to the identification of cardiac glycosides such as digoxin. Digoxin was tested in a clinical phase II trial (completed in July 2018) for breast cancer treatment (see clinicaltrials.gov). However, the use of glycosides in cancer therapy has been criticised for its non-specific effects on tumour and normal cells alike and potentially an increased risk for development of invasive cancer. Semenza has remained at the Johns Hopkins where he is the founding director of the Vascular Programme at the Johns Hopkins Institute for Cell Engineering.

## Peter J. Ratcliffe


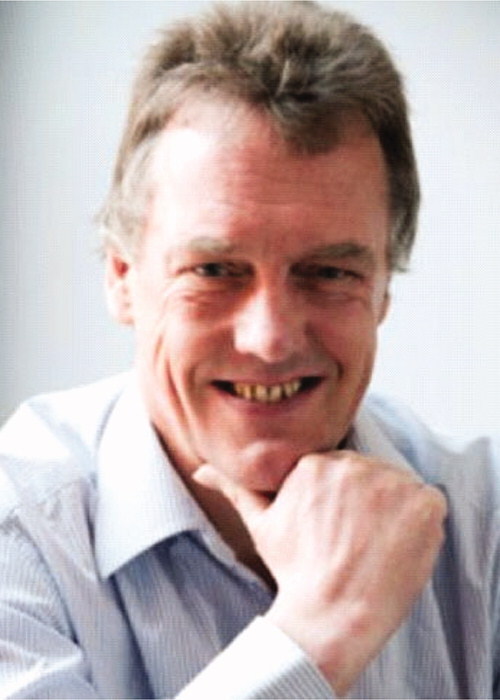


Peter J. Ratcliffe received his MD from the University of Cambridge and St Bartholomew’s Hospital, London, in 1978. He relocated to Oxford University, where he trained in renal medicine, with a particular focus on renal oxygenation. Ratcliffe has been a practicing clinician at the John Radcliffe Hospital, Oxford, and has been a Nuffield Professor of Clinical Medicine and head of the Nuffield Department of Clinical Medicine at the University of Oxford since 2004. Since 2016 he has been the director of the Target Discovery Institute, University of Oxford, and Clinical Research Director at the Francis Crick Institute.

In 1990, then a Wellcome Trust Senior Fellow, Ratcliffe set up the Hypoxia Biology laboratory in the Weatherall Institute of Molecular Medicine, Oxford. His focus on EPO led him on to study EPO transcription in different tissues. He discovered that many different cell types could switch on several orders of magnitude higher expression of EPO and other genes when deprived of oxygen (25,26). This was dependent on a 3′ enhancer in the EPO gene, the HRE, that several groups, including Ratcliffe’s, had identified (9). A 9-nucleotide stretch was later identified in the phosphoglycerate kinase 1 and lactate dehydrogenase A genes and shown to be present in and confer the same properties as the EPO gene 3′ enhancer (27).

Ratcliffe’s group went on to dissect the structural features of HIF-1α with regard to oxygen regulation and identified a sequence conferring sensitivity to regulation by hypoxia, cobalt ions, or iron chelation, which they suggested mediates regulation through protein stability (28). In their 1999 hallmark paper in *Nature*, a critical role for the von Hippel-Lindau (VHL) tumour suppressor (pVHL) in HIF-1 regulation was identified (29). Ratcliffe showed the critical, direct complex between pVHL and HIF-1, demonstrating that in VHL-defective cells HIF-1α is constitutively stabilised and activated. Re-expression of VHL restored oxygen-dependent instability (29).

Soon after, the Ratcliffe and Kaelin groups published back-to-back in *Science* that prolyl hydroxylation is the crucial oxygen- and iron-dependent posttranslational modification of HIF-1α, required for recognition by pVHL (30,31). Both studies identified the critical Pro^564^, conserved in HIF-1α throughout phylogeny. Further, Jaakkola et al. extrapolated from the known properties of prolyl-4-hydroxylase to predict that the HIF-PH uses O_2_ as a substrate and 2-oxoglutarate and Fe(II) as cofactors, where Fe(II) can be substituted for by cobalt(II), leading to enzymatic inhibition (31). A second, independent, prolyl hydroxylation site in HIF-1α was identified by Ratcliffe soon after (32).

Both the Kaelin and Ratcliffe groups contributed in different collaborative efforts to solve the structural aspects of how pVHL recognises hydroxylated proline residues in HIF-1α to cause its degradation, showing that the hydroxyproline inserts into a gap within the pVHL hydrophobic core. Interestingly, this pVHL gap is a mutational hotspot in cancer (33,34). With an amazing productivity, Ratcliffe soon thereafter published another hallmark paper, for the first time identifying HIF prolyl hydroxylases (PHDs) (35). Here, in an outstanding contribution from the Ratcliffe lab, Epstein et al. presented a novel class of prolyl hydroxylases in mammalian cells, targeting HIF-1α directly in a manner modulated by oxygen sensing. The foundation for their work was the identification of the *C. elegans egl-9* gene product encoding a 2-oxoglutarate-dependent dioxygenase as the prolyl hydroxylase for a HIF-1α homolog in *C. elegans* (35). The identification of *egl-9* allowed sequence and predicted secondary structure comparisons with mammalian dioxygenases.

Ratcliffe has thereafter focussed his research activities on the extent, mechanisms, and biological functions of prolyl hydroxylases and related oxygenases with a focus on pharmacological manipulation (see, for example, 36–38).

## William G. Kaelin


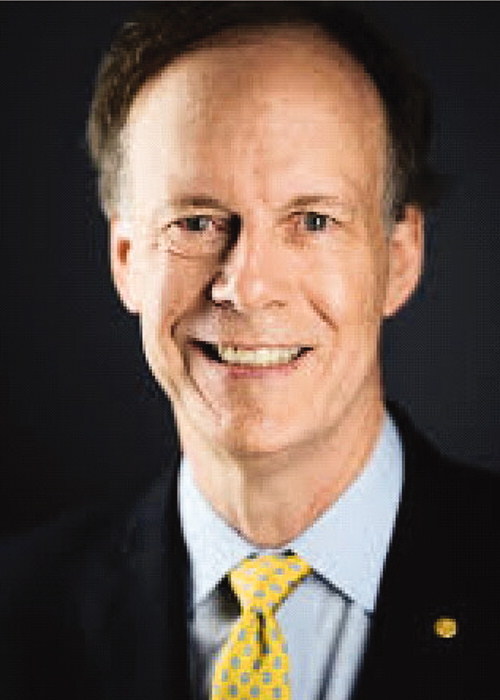


William G. Kaelin Jr received his bachelor degree in mathematics and chemistry and subsequently his MD at Duke University where he graduated in 1982. He did his residency at Johns Hopkins but went on to the Dana Farber Cancer Institute in Boston where he joined David Livingston to study tumour suppressors, in particular the retinoblastoma tumour suppressor. In 2002, he became a professor at Harvard Medical School.

While retinoblastoma was a major research interest in Livingston’s lab, Kaelin soon focussed on another tumour suppressor gene, pVHL. In 1995, when he had established his own lab, Kaelin and co-workers published the full-length sequence of the pVHL tumour suppressor, a 30-kDa cytoplasmic protein (39). In 1996, a very important step was taken towards the connection between pVHL and oxygen sensing (40). An important observation in this study was that pVHL inhibited production of vascular endothelial growth factor (VEGF), glucose transporter 1 (GLUT1), and platelet-derived growth factor B (PDGFB). As VEGF, GLUT1, and PDGFB had been shown by—among others—Ratcliffe (41) to be associated with HIF, the connection between pVHL and HIF was becoming apparent.

Kaelin’s lab went on to show that a frequently mutated region of pVHL can bind to ubiquitin ligase complexes (42). Subsequently Kaelin showed that pVHL is required for HIF-1 ubiquitination and that the pVHL-ubiquitin ligase complex binds directly to the oxygen-dependent domain of HIF-1α, the region targeted for ubiquitination (43). This important insight was validated and extended almost simultaneously by several groups (Ratcliffe, Poellinger, Conaway; for a review, see 44). Kaelin concluded that the ‘vascular tumours that characterise VHL disease may be caused by inappropriate accumulation of HIF under normoxic conditions, leading to overproduction of angiogenic peptides such as VEGF’ (43). Kaelin, in collaboration with other groups, then solved the structure of the pVHL–ubiquitin ligase complex (45).

Very soon thereafter, the Ratcliffe and Kaelin groups published their papers back-to-back in *Science*, demonstrating that prolyl hydroxylation is the crucial oxygen- and iron-dependent posttranslational modification of HIF-1α, required for recognition by pVHL and HIF-1 stabilisation (30,31). Both the Kaelin and Ratcliffe groups contributed in different collaborative efforts to solve the structural aspects of pVHL recognition of HIF-1α prolyl hydroxylation (33,34); however, Ratcliffe was first to publish on the identification of a HIF prolyl hydroxylase (35). Kaelin followed suit and identified human EGLN1 (the homologue of *C. elegans egl-9*) as a prolyl hydroxylase binding to and hydroxylating HIF-1α peptides (46).

After this rapidly developing phase in the understanding of oxygen sensing and the role in physiology and disease, Kaelin has continued to investigate hypoxia regulation and therapeutic applications in the treatment of kidney cancer, as *VHL* mutation or hypermethylation is very common in sporadic renal cell carcinomas. He has used genetic models to address the contribution of HIF-2α, compared to HIF-1α, in hypoxia regulation in the skin and the liver (47) and subsequently presented the effects of an HIF-2α antagonist, PT2399, in preclinical kidney cancer models (48).

Other interesting papers of relevance to cancer from Kaelin’s group include the finding that glutamate, secreted from triple-negative breast cancer, promotes cysteine depletion. The therapeutic potential of targeting dysregulated glutamine metabolism in different human cancer forms including breast cancer has been addressed by many. However, Kaelin and co-workers showed that PDH2 (EGLN1) undergoes oxidative self-inactivation in the absence of cysteine, allowing HIF-1α stabilisation (49). Kaelin has also addressed the effect of PDH2 inhibition/HIF stabilisation in protection against myocardial ischemia-reperfusion injury, which involves α-ketoglutarate-dependent production of hepatic kynurenic acid that mediates cardiac ischaemic protection (50).

## HIF-based cancer therapeutics

By now, it is an established dogma that rapid-growing solid tumours with time develop a hypoxic centre into which infiltrating inflammatory and immune cells are attracted. The phenotype of the inflammatory/immune cells may be protumoral, acting to support tumour growth, for example by producing VEGF, inducing tumour angiogenesis (51). The vessels in the tumour are, however, as a rule dysfunctional, with collapsed lumen and poor blood flow. They form rapidly but fail to stabilise. Therefore, in spite of the high VEGF production, the need for oxygenation in the tumour core is not met, hypoxia persists, and the vicious circle towards increased malignancy and spread is propagated (52–54). Although major insights have come from research on mouse models, there are overwhelming data on the increased stability of HIF-1α or HIF-2α expression in human cancer, promoting progression and worsening prognosis in human solid cancers such as breast, prostate, and colorectal cancer (55–57).

Many attempts have been made to exploit the possibility to inhibit the HIF pathway in cancer with the aim to halt growth and dissemination, boost anti-tumour immunity, and to improve the efficiency of therapeutics such as radiotherapy. One recent example of a HIF pathway drug is evofosfamide (Evo), a hypoxia-activated prodrug which promotes the release of a DNA-alkylating agent, bromo-isophosphoramide mustard. Evofosfamide recently failed in two phase III clinical trials, one focussed on pancreatic cancer and the other on advanced soft tissue sarcoma. However, clinical studies with evofosfamide continue, now in combination with immunotherapy (ipilimumab). Another recent failure is tarloxotinib bromide, a hypoxia-induced prodrug targeting the epidermal growth factor receptor.

Even though therapies directly targeting HIF this far have failed, many other strategies to exploit oxygen sensing in a tumour context are being developed. In an elegant collaborative approach Magnus Essand at Uppsala University and Claire Lewis at Sheffield University exploited the fact that hypoxic regions in cancer are accompanied by inflammation (58,59). In their approach, macrophages are transduced with regulatory elements consisting of an HRE to drive expression of E1A (the transforming gene of adenovirus) for expression of an oncolytic adenovirus. Using promoter elements from prostate-specific genes, expression of the oncolytic virus is restricted to prostate cancer epithelial cells. Macrophages transduced with these regulatory elements are injected in the circulation of mice where they infiltrate the hypoxic regions of prostate cancer. There, virus will be produced that specifically proliferates in, and thereby kills, prostate cancer cells. With this strategy, using macrophages as a vehicle, the otherwise therapeutically inaccessible hypoxic tumour core can now be reached, resulting in remarkable suppression of tumour growth and metastatic spread (58). The suppressive effect is maintained over long periods of time, resulting in prolonged survival of the mice. Whereas untreated mice succumb from their prostate cancer at day 14, mice treated with macrophages equipped with the capacity to produce oncolytic viruses survive until the end of the observation period at day 42 (58). This is indeed a remarkable effect in a mouse tumour model. The strategy to deliver oncolytic virus to the hypoxic tumour core is now being tested in clinical trials.

## Conclusions

According to Alfred Nobel’s will, the Nobel prize in Physiology or Medicine is awarded for a discovery of great benefit to human kind. In 2019 it went to Semenza, Ratcliffe, and Kaelin for their discovery of oxygen sensing, a prize of the ‘classical school’ recognising a fundamental biological principle. While the full potential with regard to cancer therapeutics still is to come, let’s enjoy the stunning beauty of the biological machinery evolved to keep us oxygenated, allowing us to grow, but only as much as is needed! However, as always, questions remain. Are there other oxygen-sensing mechanisms? Can oxygen-sensing mechanisms be highjacked by the cancer cell? How do we accurately measure hypoxia? The interested reader is referred to the excellent review by Macklin et al. (60).
